# ﻿Replacement name for a Panamic bivalve (Mollusca, Bivalvia, Cyrenidae)

**DOI:** 10.3897/zookeys.1108.83037

**Published:** 2022-06-23

**Authors:** Eugene V. Coan, Paul Valentich-Scott

**Affiliations:** 1 Santa Barbara Museum of Natural History, 2559 Puesta del Sol, Santa Barbara, CA 93105, USA Santa Barbara Museum of Natural History Santa Barbara United States of America

Thomas A. Neubauer of the Systematics & Biodiversity Lab, Justus Liebig University, Giessen, Germany, has called to our attention that *Cyrenaacuta* Prime, 1861, published in October, is a junior primary homonym of *Cyrenaacuta* Ludwig, 1861, published in January that same year.

Prime’s species, currently known as *Polymesodaacuta* (Prime, 1861: 355), ranges from Costa Rica to Ecuador, where it occurs intertidally in mangrove areas (Coan and Valentich-Scott 2012: 464–465). The holotype of this species is deposited in the Museum of Comparative Zoology, USA, Harvard University (MCZ 176951) (Johnson 1959: 441). In Prime’s original description the type locality was given only as Central America.

*Cyrenaacuta*Ludwig (1861: 197–199, pl. 72, figs 15, 16) was described from the early Miocene of Münzenberg, Hesse, Germany, where it occurs with other fresh and brackish-water species (Kadolsky 2008). The species is presently considered a junior synonym of *Falsocorbiculafaujasii* (Deshayes, 1830: 51) [originally *Cyrena*] (Ott et al. 2009). It also remains in the Cyrenidae.

The International Code of Zoological Nomenclature’s (1999) Article 23.9 [Reversal of Precedence] does not apply in that Prime’s name has been used less than ten times in the last 150 years since its publication, athough Ludwig’s name has seen little mention because it has been long regarded as a junior synonym.

**Figure 1.**
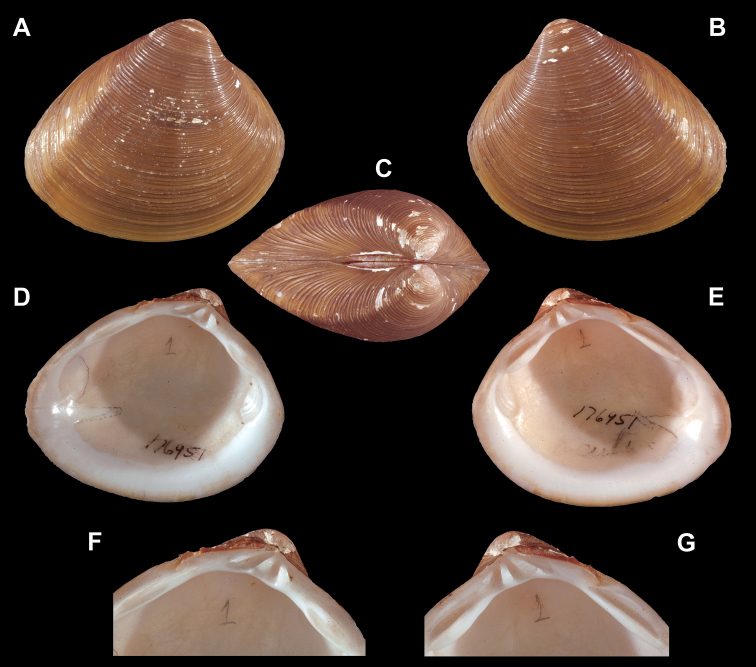
Holotype of *Cyrenaacuta* Prime, 1861, renamed herein as *Polymesodaneubaueri* nom. nov. (MCZ 176951), length 41 mm, height 35 mm. **A** exterior of right valve **B** exterior of left valve **C** dorsal view of both valves **D** interior of left valve **E** interior of right valve **F** close up of hinge of left valve **G** close up of hinge of right valve.

We hereby rename *Polymesodaacuta* as *Polymesodaneubaueri* Coan & Valentich-Scott, 2022 (Fig. 1). We restrict the type locality to Costa Rica, Guanacaste Province, Lower Río Tempisque; 10.2583°N, 85.2644°W; intertidal zone, because Prime’s locality could have been on either the Atlantic or Pacific coast of Central America and there is no additional information accompanying the holotype (International Code of Zoological Nomenclature (1999: Article 76A). Material from this locality in the Santa Barbara Museum of Natural History collection matches the holotype.
